# Evaluating the Impact of the Community-Based Health Planning and Services Initiative on Uptake of Skilled Birth Care in Ghana

**DOI:** 10.1371/journal.pone.0120556

**Published:** 2015-03-19

**Authors:** Fiifi Amoako Johnson, Faustina Frempong-Ainguah, Zoe Matthews, Andrew J. P. Harfoot, Philomena Nyarko, Angela Baschieri, Peter W. Gething, Jane Falkingham, Peter M. Atkinson

**Affiliations:** 1 Division of Social Statistics and Demography & Centre for Global Health, Population, Poverty and Policy, Faculty of Social and Human Sciences, University of Southampton, Highfield, Southampton, United Kingdom; 2 Regional Institute for Population Studies, University of Ghana, Legon, Accra, Ghana; 3 GeoData Institute, University of Southampton, Highfield, Southampton, United Kingdom; 4 Ghana Statistical Service, Head Office Building, Accra, Ghana; 5 Spatial Ecology and Epidemiology Group, Tinbergen Building, Department of Zoology, University of Oxford, South Parks Road, Oxford, United Kingdom; 6 Geography and Environment, University of Southampton, Highfield, Southampton, United Kingdom; Centers for Disease Control and Prevention, UNITED STATES

## Abstract

**Background:**

The Community-based Health Planning and Services (CHPS) initiative is a major government policy to improve maternal and child health and accelerate progress in the reduction of maternal mortality in Ghana. However, strategic intelligence on the impact of the initiative is lacking, given the persistant problems of patchy geographical access to care for rural women. This study investigates the impact of proximity to CHPS on facilitating uptake of skilled birth care in rural areas.

**Methods and Findings:**

Data from the 2003 and 2008 Demographic and Health Survey, on 4,349 births from 463 rural communities were linked to georeferenced data on health facilities, CHPS and topographic data on national road-networks. Distance to nearest health facility and CHPS was computed using the closest facility functionality in ArcGIS 10.1. Multilevel logistic regression was used to examine the effect of proximity to health facilities and CHPS on use of skilled care at birth, adjusting for relevant predictors and clustering within communities. The results show that a substantial proportion of births continue to occur in communities more than 8 km from both health facilities and CHPS. Increases in uptake of skilled birth care are more pronounced where both health facilities and CHPS compounds are within 8 km, but not in communities within 8 km of CHPS but lack access to health facilities. Where both health facilities and CHPS are within 8 km, the odds of skilled birth care is 16% higher than where there is only a health facility within 8km.

**Conclusion:**

Where CHPS compounds are set up near health facilities, there is improved access to care, demonstrating the facilitatory role of CHPS in stimulating access to better care at birth, in areas where health facilities are accessible.

## Introduction

In 2008, the Ghana Minister of Health declared maternal mortality a national emergency due to the slow progress in reducing the high level of maternal mortality in the country [1]. Since then successive governments have been explicit about their intent to address the problem. For example, at the 2010 African Union Head of States Conference in Uganda, the (then) President of the Republic of Ghana, President John Evans Atta Mills stated that “no woman should die while giving life”. His successor, President John Dramani Mahama, at the 2012 presidential elections debate, promised to continue action to reduce the high rate of maternal mortality in the country. One of his stated solutions was the expansion of the Community-based Health Planning and Services (CHPS) initiative to bring healthcare to the doorsteps of women in rural areas. This was affirmed in his party’s (National Democratic Congress) election manifesto, which identified CHPS as a priority health initiative to improve access to health care for all [2].

The commitment to reduce Ghana’s high maternal mortality has become crucial, as recent estimates show that maternal deaths remain unacceptably high and progress continues to be slow. Ghana’s maternal mortality ratio of 350 per 100,000 live births in 2010 represents a 40% decline between 1990 and 2010 [3]. Although this suggests that Ghana is making progress, the rate of reduction (2.6% per year) is insufficient to attain the national MDG target of 185 per 100,000 live births by 2015.

More recently, service delivery indicators give a hopeful picture of positive trends. The percentage of births attended by skilled health personnel has increased from 47% in 2003 to 68.4% in 2011 [4, 5]. However, although almost all women seek antenatal care from a health professional and four in five women seek postnatal care, less than half of all women receive all three maternity care components (antenatal care, care at birth and postnatal care) from a skilled provider [5]. Moreover, uneven progress has led to widening inequalities [6]. The 2010 Emergency Obstetric and Newborn Care (EmONC) assessment revealed that only one third of pregnant women requiring emergency care actually receive it [7]. Although the proportion of women attended by a skilled health professional at birth has increased, the gap between the regions with the largest and smallest proportions has widened over time [7]. Poor staff attitudes and unsatisfactory facilities sometimes involving long and expensive journey times have been identified as key factors affecting women’s choice to give birth in a facility, next to cultural and other barriers [5, 8].

The CHPS framework is based on two experiments conducted in the Kassena-Nankana and Nkwanta districts of the Upper East and Volta regions, respectively [9]. These experiments demonstrated that engaging a resident nurse in a community and involving traditional leaders and community members in the provision and management of healthcare substantially reduces childhood mortality, builds male participation in family planning and improves health system accountability [10–12]. In 1999, a policy statement leading to the launch of the CHPS initiative was adopted at a National Health Forum convened by the Ghana Ministry of Health. A key attraction of the initiative was that it could be replicated across the country, particularly in rural communities with modest resources [9].

Since its inception, CHPS has become a major national programme focused on promoting primary health care in marginalised communities. Following a slow start, the number of functional CHPS compounds doubled from 868 to 1675 between 2009 and 2011 [13]. The programme relies on community resources for construction, service delivery and programme oversight [7]. As such, it represents a national mobilisation of grass-roots action, resources and leadership in promoting quality health care in marginalised communities. With a primary focus on deprived rural communities, CHPS aims to provide essential primary health care services and health education within demarcated geographic areas referred to as CHPS zones, which are staffed by resident Community Health Officers and supported by community volunteers, community health committees and traditional health care providers including native doctors, Traditional Birth Attendants (TBAs) and herbalists [14]. CHPS activities cover educational programmes aimed at prevailing health problems, prevention and care, improving physical wellbeing and personal hygiene, maternal, newborn and child health care including antenatal and postnatal care as well as family planning and immunisation [14].

Although research evidence shows that skilled birth care is essential in addressing the high rates of child and maternal mortality and morbidity in low and middle-income countries, provision of skilled delivery care is not stated as a core activity in the operational policy of CHPS [14, 15]. However, the initiative is one of the major services prioritised to improve maternal and child health [16]. The strategic goal is that the initiative will promote skilled delivery attendance through community participation, promotional activities and referral services [14, 17]. For example, Ghana’s MDG 5 Acceleration Framework (MAF) operational plan emphasised the need for expansion of CHPS zones to address geographical access and remove financial and cultural barriers to skilled maternity care by encouraging communities to use maternity facilities with midwives and other professional health workers that are trained to deal with childbirth [7].

Despite the focus on CHPS as an intervention to accelerate progress to MDG-5 and stimulate the attainment of the post-MDG agenda of equitable health outcomes, strategic intelligence on the impact of the CHPS initiative is lacking while continued problems persist in relation to geographical accessibility to care for rural women [8]. The initiative was particularly designed for the Ghanaian health system, but has parallels elsewhere; for example the ‘bidan di desa’ midwife health posts in Indonesia [18], and the H4+ initiative in Afhganistan to train and deploy locally approved midwives [19]. Other programmes include the Somalia Basic Development Needs Initiative, the Malawi Health Surveillance Assistants programme, and the Husband Schools in Niger and Cote d’Ivoire [20–23]. However, this and other initiatives to reduce geographical barriers to care have focused on interim measures to provide a minimally trained assistant, rather than a professional nurse or midwife. Examining the impact of these initiatives, especially those that have embedded professional care such as the CHPS scheme is crucial for accelerating progress to MDG-5 and beyond.

The aim of this paper is therefore to investigate the growth of CHPS compounds in rural communities and map their influence on facilitating skilled birth care in rural communities. Using modern GIS techniques, it is now possible to link the geographical location of these functional CHPS compounds with contemporaneous data on skilled care at birth to assess the success of the initiative in promoting use of services in rural areas. By doing this it is possible to directly investigate whether the CHPS compounds serve as a facilitator for skilled birth attendance to occur in local hospitals.

Given the urgency to accelerate progress towards 2015 under the new MAF operational plan, such analyses are vital. Previous research on progress in MDG-5 has focused on indicators at the country level. However, there are increasing concerns of widening inequalities at the sub-national level [24]. Research evidence shows that even where national maternal and newborn mortality rates have declined, there are subgroups where survival rates and access to services have not changed or even worsened over time [25–28].

Despite an encouraging trend of increased interest in applying powerful GIS technology for improving maternal and newborn health outcomes in recent years, the literature on geographic access to maternal health services remains sparse. Within the emerging evidence, different GIS techniques have been used to map, extract or model maternal and newborn health outcomes [8, 29, 30]. This paper builds on the literature by applying GIS techniques to examine the impact of proximity to CHPS on the uptake of skilled birth care in rural Ghana. In this study, proximity is measured as the road network distance from the centroid of a Primary Sample Unit (PSU) also referred to as Census Enumeration Areas (EA) to the nearest conversional health facility and also to the nearest CHPS referral point. Calibration of the road network distance is discussed in subsequent sections.

## Methods

### Data

The data for the analysis come from the two most recent rounds (2003 and 2008) of the Ghana Demographic and Health Surveys (GDHS), a geo-referenced database of health facilities and digitised topographic database of national road networks from a national programme of land surveillance. The land surveillance was conducted by the Water Research Institute, the Centre for Remote Sensing and Geographic Information Services (CERSGIS) of the University of Ghana, Department of Feeder Roads, Ghana Survey Department and the Forestry Commission of Ghana between 1995 and 2005. The outcome variable of interest and controls were derived from the GDHS, whilst the main predictor of interest (distance to CHPS and health facilities) were derived from the geo-referenced database of health facilities and national road networks. Description of the datasets, variables and calibration of distances are discussed below.

#### Demographic and Health Surveys

The 2003 and 2008 GDHS are nationally representative cross-sectional surveys of women and men aged 15–49 years and 15–59 years, respectively. The surveys collected demographic and health information on women, men, children and other members of households. Information on where a woman gave birth and who attended the birth was collected for all births five years preceding each survey. The births recorded in the two surveys cover the period July 1999 to October 2008, which coincide with the launch of the CHPS initiative in 1999. Prior to 2005, CHPS was a rural policy [11, 31]. In 2005, the Government of Ghana, the Ghana Ministry of Health and Ghana Health Service adopted CHPS as national policy with the aim of developing urban health systems in marginalised urban communities [14, 31]. However, due to the slow implementation of CHPS in urban areas, only two pilot urban CHPS compounds (in Tema and Glefe, both in the Greater Accra region) supported by the CHPS Technical Assistance Project of the USAID were functional [32] within the study period. The analysis is, therefore, restricted to rural communities.

The outcome variable of interest focuses on the proficiency in the skills of the attendant at birth. Skilled attendant is used to refer exclusively to people with midwifery skills (e.g. doctors, midwives, nurses), trained in the skills necessary to manage normal deliveries, diagnosis, management of complications and referrals [33]. The data cover 4349 births. The response variable was binary coded 1 if a birth was attended by a skilled professional and 0 otherwise. To ensure minimal recall bias, we examined the consistency between reported place of birth and type of birth attendant. Non-institutional births reported to have been attended by skilled professionals constitute less than 0.05% and were excluded from the analysis. The choice of control variables was based on literature and data availability. The selected control variables were maternal age and education, ethnicity, religion, parity, number of antenatal visits, partner’s educational status, household wealth status, and region of residence.

#### Geospatial database of health facilities and CHPS

A national georeferenced database of health facilities providing care at birth and digital topographic data on road networks were used as input to a network analysis algorithm to calculate the distance from each PSU in the GDHS to the nearest health facility and also CHPS referal point, often referred to as a CHPS compound. The calibration of network distance to nearest health facility and CHPS coumpound is discussed in the subsequent section.

The list of health facilities was compiled from three sources: a list of 2021 health facilities obtained from the Ghana Ministry of Health, a web resource of health facilities maintained by the Ghana Mininistry of Health and a georeferenced list of 1915 health facilities compiled by CERSGIS, University of Ghana. The lists were cross-checked and reconciled. Facilities without latitude and longitude values were georeferenced manually by matching with facility and town names on Google Earth. In unresolved cases, we contacted district health offices to confirm locations. Facilities such as psychiatric hospitals, supplementary feeding centres, nursing training colleges and administrative offices which do not offer maternity services were excluded. Where there was more than one facility at a single location (either because they shared the same building or because they were georeferenced using a village location) the highest order facility was retained for subsequent analysis, thus, avoiding duplications. In all there were 1688 georeferenced conventional health facilities throughout the country.

The EmONC audit was used to classify the EmONC status of the facilities based on the signal-functions they offer [7, 34]. Facilities offering all nine signal functions including availability of blood transfusion and surgical/caesarean section capability (76 in all) were classified as comprehensive-EmONC facilities [7]. Facilities offering between six and eight signal functions (81 in all) were classified as partial-EmONC. Since uptake of maternity care in Ghana is often between partially functioning EmONC which are able to respond appropriately to a range of birth complications and non-EmONC [8], we classfied the facilities into two groups—EmONC facilities (6 or more signal functions) and non-EmONC (less than six signal functions).

CHPS covers a set defined catchment areas referred to as CHPS zones. The recommended population covered by a CHPS zone is 3000 to 4500 [14]. A CHPS zone may cover one or more villages or communities. Some CHPS zones have a physical structure purposely built or designated for providing CHPS services, referred to as CHPS compounds and staffed by Community Health Officers. The CHPS compound is located in one of the communities within the zone. In such situations, the CHPS compound serves as the referral point. In CHPS zones where there is no physical structure designated for CHPS activities, the location or residence of the Community Health Officer in charge of the CHPS zone is used as the referral point. The GPS coordinates collated by the Ghana Ministry of Health references the location of the CHPS compounds (where there is a physical structure) and the location of the Community Health Officer (where there is no physical structure). In all, there were 458 CHPS compounds, functional in September 2008.

#### Calibration of network distance to nearest health facility and CHPS

The road network data were modified to incorporate ferry routes connecting settlements on the shores of the Volta lake. The distance between the location of each PSU and the nearest health facility and CHPS referral point was calculated using the ‘closest facility’ functionality in ESRI ArcGIS Network Analyst software. For the routing algorithm to function correctly, the national road network dataset had to be topologically cleaned, ensuring that the segments of the network were connected appropriately prior to undertaking the analysis. The topological checking and cleaning was also carried out in the ESRI ArcGIS software. All distance calculations were carried out using the Ghana Metre Grid projected coordinate system (EPSG code 25000). For the road network analysis, all georeferenced facilities were included irrespective of the rural-urban location, since the closest facility for some rural communities was in an urban location.

**Fig 1:**
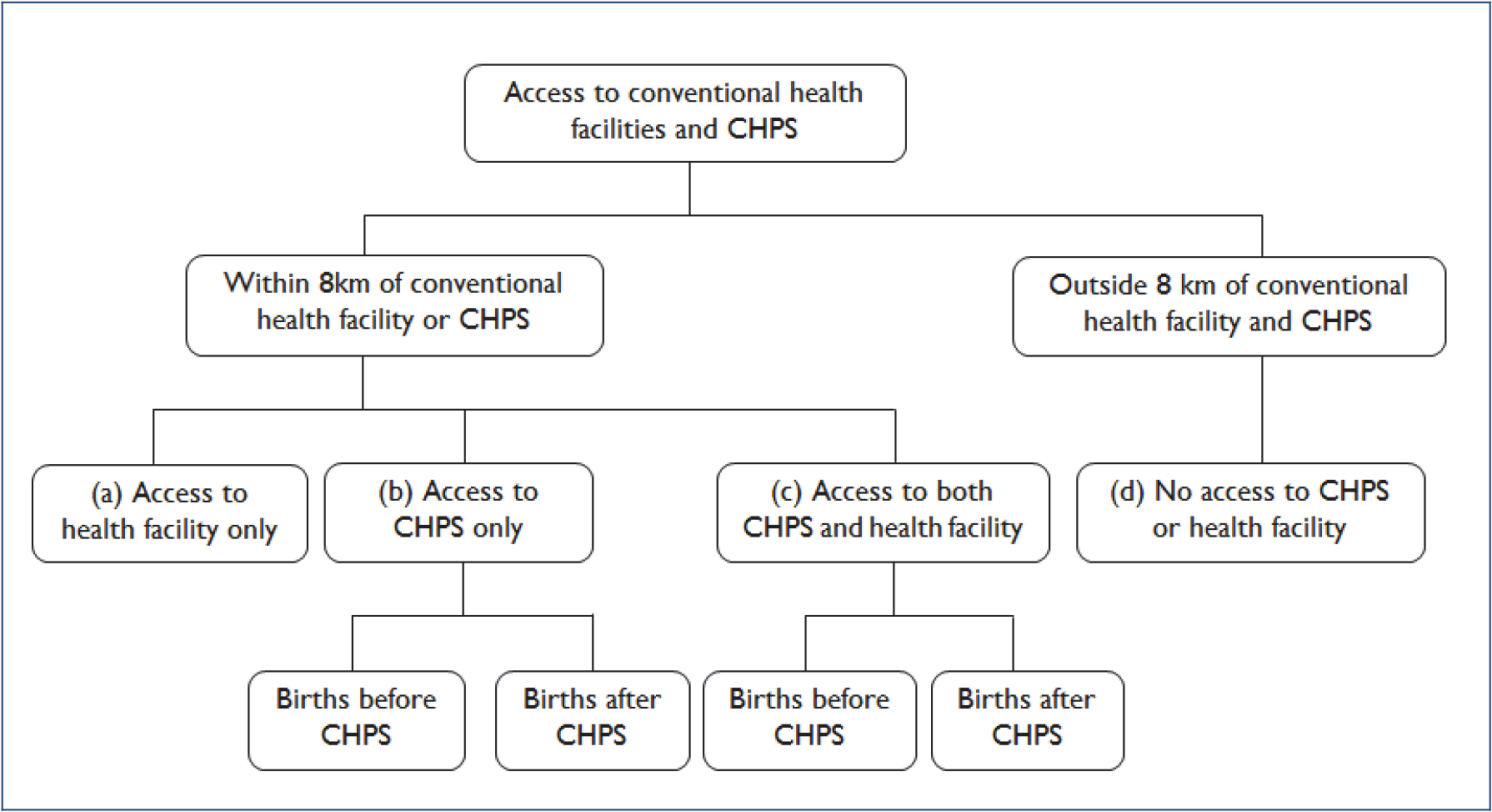
Conceptualisation of access to CHPS and conventional health facilities.

Since many health facilities and CHPS compounds were not located exactly on the nearest road segment, all community locations and health facilities were assigned to the closest location on the road network measured as a straight line distance, where that distance was less than 5 km, as shown in the sample caption of Fig. 1, labels A and B. The reported distance was a sum of the straight line distances between the community and nearest road segment, the along road distance, and the straight line distance between the road and the facility. Twenty-six of the 463 communities and 36 of the 1688 health facilities were found to be more than 5 km from the nearest road. In this case, the straight line distance to the nearest facility was used (Fig. 1, label C). In this study, topographic obstacles were not taken into account in the calibration of the distances. For each community, the closest distance to health facility and CHPS referral point was calculated.

**Fig 2:**
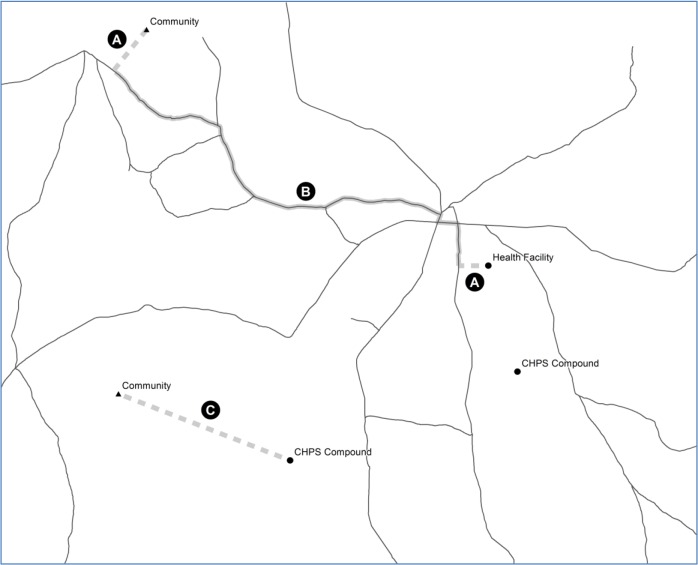
Calibration of distance to health facilities and CHPS referral points.

Based on the Ghana Health Service policy framework which defines access as being within 8 km of a facility [35, 36], all communities were classified into those within 8 km of—(a) health facility only, (b) CHPS only, (c) both a health facility and CHPS and (d) outside 8 km of both a health facility and CHPS. Thus, in this study access to a health facility or CHPS is defined as being within 8 km of the referral point as stipulated by the Ghana Health Sevice policy framework. For CHPS, the Ghana Ministry of Health provided the month and year they became functional. Information on when conventional health facilities became functional were not available. Relating the month and year of CHPS becoming functional in the communities to the date of birth of the children in the survey which was available from the GDHS, births in (b) and (c) were futher disaggregated into those that occurred before and after CHPS become functional. Operationalisation of access to health facilities and CHPS is illustrated in Fig. 2.

### Statistical analysis

The distribution of uptake of skilled care at birth by access to CHPS compounds and health facilities and wealth status were examined through bivariate analysis. Chi-squared tests were used to investigate significant differences. Multilevel logistic regression techniques were used to examine the adjusted effect of access to CHPS and health facilities on the uptake of skilled care at birth, accounting for potential confounders and clustering of the data. Two-level binary logistic regression models were used with children nested within communities (PSUs) to model uptake of skilled delivery care allowing for between-community homogeneity to be examined. There were 4349 children (level 1) nested in 463 communities (level 2). We conducted additional analysis, separately for communities where CHPS have become functional within 8 km but access to health facilities remain limited (n = 511, PSUs = 51) and also where CHPS has supplemented health facilities within 8 km (n = 502, PSUs = 57) to further examine the before and after impact of CHPS on skilled maternity care uptake in those communities where CHPS has become functional. The longitudes and latitudes of the primary sampling units were mapped using the ArcGIS software to identify duplicated communities.

A sequential model-building process was used to examine the extent to which access to CHPS, health facilities and the intermediate factors explained uptake of skilled care at birth between communities. The rationale for adopting a sequential model-building process was to investigate how the association between distance to a facility and uptake of skilled birth care changed when other important intermediate factors were included in the model. Model 1 controlled for the between-community differences to account for the hierarchical structure of the data and year of survey to account for the survey effect. Model 2 added the primary variable (access to health facility and CHPS). Model 3 further added the intermediate factors and Model 4 included the region of residence. At each stage of the model-building process variables that were not significant at p<0.05 were discarded. The significance of these variables was further tested in the final model. The models were fitted using MLwiN 2.26 [37]. The Penalized Quasi-Likelihood (PQL) estimation procedure with a second-order Taylor series approximation was used to estimate the model parameters [38–40].

## Results

### Bivariate analysis

**Fig 3:**
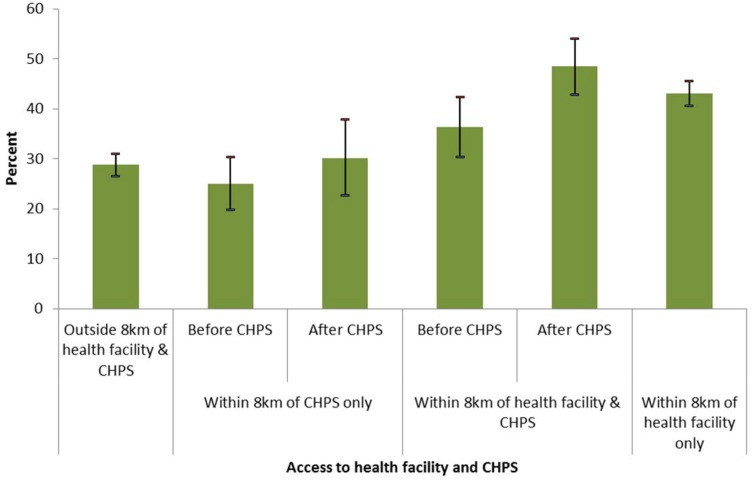
Distribution of skilled birth care by access to health facilities and CHPS for births that occurred before and after CHPS became functional, with 95% confidence interval.

The analysis revealed that CHPS coverage was very limited in rural Ghana. Only 9.9% of all the births were in communities within 8 km of CHPS. The majority of births either occurred in communities with access to a health facility (42.9%) or those with no access to both health facility and CHPS (47.2%). Fig. 3 shows the distribution of skilled birth care by access to health facility and CHPS compound. Overall, 35.5% of all rural births (30.9% and 41.8% for the 2003 and 2008 GDHS, respectively; *p<0*.*001*) were attended by skilled health personnel. There exist significant differences (*p<0*.*001*) in uptake of skilled birth care by access to health facility and CHPS. Where there was no access to both health facility and CHPS, 28.8% of births were attended by skilled attendants compared to 43.0% for those in communities with access to a health facility (Fig. 3). The results also suggest that uptake of skilled care at birth has increased in communities where CHPS has become functional. In communities where CHPS has become functional but access to health facilities remain limited, uptake of skilled birth care was 25.0% prior to CHPS becoming functional but increased to 30.1% after CHPS became functional (*p = 0*.*134*), suggesting that the increase is not large enough to be statistically significant. In communities where health facilities have been supplemented with CHPS within 8 km, uptake of skilled birth care increased from 36.4% to 48.5% when CHPS became functional (*p = 0*.*005*), indicating a significant increase.

**Fig 4:**
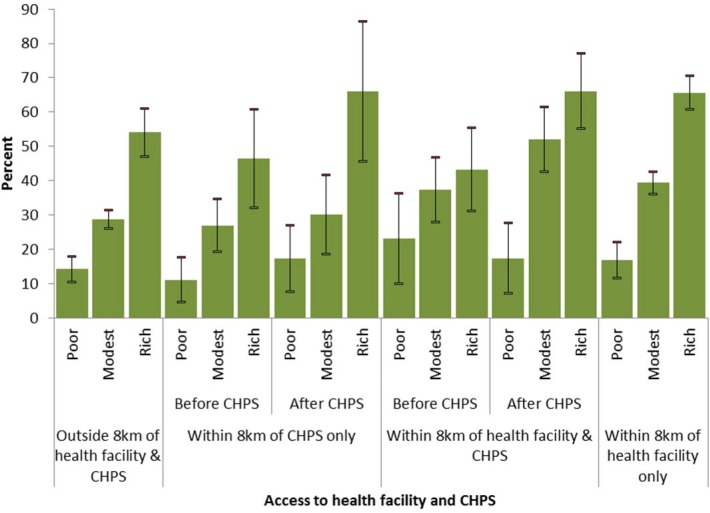
Household wealth differentials in the distribution of skilled birth care by access to health facilities and CHPS for births that occurred before and after CHPS became functional, with 95% confidence interval.

Fig. 4 shows the distribution of uptake of skilled care at birth by access to health facility, CHPS and household wealth status. The results demonstrate that access to health facility as well as wealth status significantly (*p<0*.*001*) influence uptake of skilled birth care (Fig. 4). The results reveal clearly that in rural areas of Ghana, births to women from rich households are more likely to be attended by skilled health personnel when compared to those from poor households, even in communities with no access to health facility or CHPS. Also, the benefits of a CHPS compound becoming functional within 8 km accrued more to the rich when compared to the poor. For example, in communities where there was no health facility but CHPS has become functional, skilled birth care increased by 5.8% (from 11.1% to 16.9%; p = 0.153) for the poor compared to 19.9% (from 46.8% to 66.7%; p = 0.065) for the rich. Where there was a health facility and CHPS also became functional, skilled birth care declined by 5.5% (from 22.5% to 17.0%; p = 0.252) for the poor, whereas there was a 22% (from 43.8% to 65.8%; p = 0.010) increase for the rich.

### Multivariate analysis

Odds ratios from the two-level binary regression analyses are presented in Table 1, along with their 95% confidence intervals. Table 1 reveals that access to health facilities and CHPS have a significant impact on the uptake of skilled birth care. Other factors which significantly influence uptake of skilled birth care include maternal age and education, religious affiliation, parity, antenatal care visits, partner’s education and region of residence. The type of facility and the EmONC status of the nearest facility were not significantly associated with uptake of skilled birth care. We tested for interaction between household wealth status and access to health facilities and CHPS, but the effects were not significant at the 5% level. This indicates that the effect of access on uptake of skilled birth care in rural areas of Ghana is independent of household wealth status.

**Table 1:** Estimated odd ratios of uptake of skilled birth care and their 95 percent confidence intervals.

	Model 1	Model 2	Model 3	Model 4
	OR [95% CI]	OR [95% CI]	OR [95% CI]	OR [95% CI]
**Control variable**				
Year of survey				
2003	1.00	1.00	1.00	1.00
2008	1.61 [1.41, 1.84]**	1.59 [1.38, 1.83]**	1.48 [1.26, 1.73]**	1.50 [1.28, 1.77]**
**Primary factor**				
Access to health facility				
No CHPS or HF		1.00	1.00	1.00
CHPS only (before CHPS)		0.90 [0.67, 1.22]	0.99 [0.71, 1.38]	0.93 [0.66, 1.32]
CHPS only (after CHPS)		0.86 [0.59, 1.25]	1.12 [0.73, 1.71]	1.14 [0.73, 1.75]
CHPS & HF (before CHPS)		1.51 [1.11, 2.05]**	1.16 [0.82, 1.65]	1.19 [0.83, 1.71]
CHPS & HF (after CHPS)		2.01 [1.52, 2.67]**	1.59 [1.14, 2.22]**	1.51 [1.07, 2.13]*
HF only		1.88 [1.61, 2.19]**	1.42 [1.20, 1.69]**	1.35 [1.13, 1.61]**
**Intermediate factors**				
Maternal age (years)				
less than 20			0.46 [0.31, 0.68]**	0.46 [0.31, 0.68]**
20–34			0.80 [0.64, 0.99]*	0.79 [0.64, 0.98]*
35+			1.00	1.00
Educational status				
No formal education			0.58 [0.47, 0.72]**	0.63 [0.51, 0.79]**
Primary			0.59 [0.49, 0.73]**	0.61 [0.50, 0.74]**
Secondary or higher			1.00	1.00
Religion				
Christian			1.00	1.00
Moslem			0.89 [0.70, 1.13]	1.04 [0.81, 1.35]
Other			0.68 [0.52, 0.88]*	0.69 [0.53, 0.91]*
Parity				
First			1.77 [1.37, 2.30]*	1.79 [1.37, 2.33]**
Second—third			0.95 [0.78, 1.15]	0.95 [0.78, 1.16]
Fourth or higher			1.00	1.00
Number of antenatal visits				
No antenatal visits			0.32 [0.26, 0.40]**	0.34 [0.27, 0.43]**
1–3 visits			0.18 [0.14, 0.24]**	0.19 [0.14, 0.25]**
4–6 visits			0.44 [0.35, 0.55]**	0.45 [0.36, 0.57]**
7 or more visits			1.00	1.00
Partner's educational status				
No formal education			0.51 [0.41, 0.62]**	0.53 [0.43, 0.65]**
Primary			0.74 [0.57, 0.96]*	0.72 [0.55, 0.93]*
Secondary or higher			1.00	1.00
Wealth status				
Poor			0.32 [0.24, 0.43]**	0.33 [0.24, 0.45]**
Modest			0.55 [0.45, 0.66]**	0.52 [0.43, 0.64]**
Rich			1.00	1.00
**Spatial factors**				
Region				
Northern				1.00
Western				2.02 [1.37, 3.00]**
Central				1.37 [0.92, 2.04]
Greater Accra				1.54 [0.87, 2.71]
Volta				1.96 [1.33, 2.88]**
Eastern				2.05 [1.37, 3.06]**
Ashanti				2.21 [1.54, 3.18]**
Brong Ahafo				3.05 [2.10, 4.44]**
Upper West				2.64 [1.72, 4.06]**
Upper East				1.78 [1.16, 2.72]**
**Random effect estimates**				
Community variance [95% CI]	1.80 [1.47, 2.13]**	1.65 [1.33, 1.96]**	0.88 [0.66, 1.10]**	0.78 [0.58, 0.98]**

**p<0.01

*p<0.05

HF—Health Facility; CHPS—Community-based Health Planning and Service

After controlling for the important predictors in the model, the results show that in communities where CHPS has become functional within 8 km but access to health facilities remain limited, uptake of skilled birth care before (OR = 0.93, 95% CI = 0.66, 1.32) and after (OR = 1.14, 0.73, 1.75) CHPS becoming functional are not significantly different from communities where access to both health facilities and CHPS remain limited (Table 1). Further analysis focusing only on communities where CHPS has become functional within 8 km, but without access to health facilities within 8 km confirmed the above results (Table 2, Model 1). The results presented in Table 2 shows that although the odds of uptake of skilled maternity care (OR = 1.40, 95% CI = 0.61, 3.24) has increased in these communities after CHPS became functional within 8 km, the increase is not large enough to be statistically significant.

**Table 2:** Estimated odd ratios of uptake of skilled birth care and their 95 percent confidence intervals for (Model A) communities where CHPS has become functional within 8 km but no conventional health facility exists and (Model B) communities where CHPS has supplemented health facilities.

	Model 1: Communities not within 8 km of health facility	Model 2: Communities within 8 km of health facility
	OR [95% CI]	OR [95% CI]
Year of survey		
2003	1.00	1.00
2008	1.03 [0.42, 2.56]	1.18 [0.69, 2.01]
Primary factor		
Timing of birth		
Before CHPS	1.00	1.00
After CHPS	1.40 [0.61, 3.24]	1.56 [1.04, 2.36]*
Intermediate factors		
Maternal age (years)		
less than 20	1.14 [0.33, 3.95]	0.72 [0.23, 2.26]
20–34	1.50 [0.76, 2.95]	0.84 [0.44, 1.61]
35+	1.00	1.00
Educational status		
No formal education	0.60 [0.26, 1.40]	1.38 [0.65, 2.91]
Primary	0.53 [0.24, 1.16]	1.21 [0.61, 2.42]
Secondary or higher	1.00	1.00
Religion		
Christian	1.00	1.00
Moslem	0.80 [0.36, 1.80]	1.51 [0.58, 3.93]
Other	0.91 [0.41, 2.03]	0.58 [0.27, 1.25]
Parity		
First	1.94 [0.85, 4.41]	1.14 [0.51, 2.54]
Second—third	1.02 [0.54, 1.93]	0.70 [0.39, 1.24]
Fourth or higher		
Number of antenatal visits		
No antenatal visits	0.27 [0.12, 0.62]**	0.49 [0.25, 0.95]*
1–3 visits	0.23 [0.09, 0.60]**	0.57 [0.25, 1.31]
4–6 visits	0.47 [0.22, 1.02]	0.77 [0.38, 1.57]
7 or more visits	1.00	1.00
Partner's educational status		
No formal education	0.65 [0.31, 1.35]	0.31 [0.16, 0.58]**
Primary	1.11 [0.45, 2.71]	0.43 [0.20, 0.92]*
Secondary or higher	1.00	1.00
Wealth status		
Poor	0.27 [0.11, 0.66]**	0.75 [0.31, 1.83]
Modest	0.42 [0.19, 0.90]*	0.99 [0.52, 1.91]
Rich	1.00	1.00
Spatial factors		
Region		
North	1.00	1.00
Middle	0.77 [0.26, 2.26]	2.02 [0.77, 5.32]
South	1.25 [0.49, 3.19]	1.21 [0.49, 3.00]
Random effect estimates		
Community level variance [95% CI]	0.63 [0.09, 1.17]**	0.88 [0.29, 1.47]**

**p<0.01

*p<0.05

CHPS—Community-based Health Planning and Service

**Note:** Sample sizes within some of the regions were very small. In this regard, the regions were aggregated into the three geo-ecological zones of the country, with North comprising of Northern, Upper East and Upper West regions, Middle consist of Eastern, Ashanti and Brong Ahafo regions and South encompassing Greater Accra, Central, Western and Volta regions.

Considering communities where health facilities have been supplemented with CHPS, the results show that before CHPS became functional the odds of uptake of skilled birth care was not significantly different (OR = 1.19, 95% CI = 0.83, 1.71) when compared to communities where access to both health facilities and CHPS remain limited, however after health facilities were suplemented with CHPS, the odds increased by 26.9% (OR = 1.51, 95% CI = 1.07, 2.13) and the effect was statistical significant (p<0.05). Supplementary analysis limited to communities where health facilities have been supplemented with CHPS within 8 km, revealed that uptake of skilled birth care improved significantly after health facilities were supplemented with CHPS (Table 2, Model 2). The results show that the odds of skilled birth care use increased by 56% after health facilities were supplemented with CHPS within 8 km.

The results presented in Table 1, also shows that the odds of uptake of skilled birth care is significatly higher (OR = 1.35, 95% CI = 1.13, 1.61) in communities within 8 km of a health facility only, when compared to communities with no access to both health facilities and CHPS within 8 km. The results further shows that in communities where there is access to both health facility and CHPS, the odds of skilled birth care are about 16% higher when compared to communities with access to only a health facility. It is also interesting to note that in communities where CHPS has become functional within 8 km, before CHPS became functional the odds of skilled birth care was 7% lower when compare to communities with no access to both health facilities and CHPS, however when CHPS became functional within 8 km, the odds of skilled birth care increased and was 14% higher, although the increase was not large enough to be statistically significant.

The significant community level variance term shown in Table 1, Model 1 reveals that there exist signifcant differences between rural communities in the uptake of skilled birth care. Access to health facilities and CHPS explained 8.3% of the between-community differences in the uptake of skilled birth care (Model 2). When the intermediate factors (Model 3) and spatial factors (Model 4) were included in the model the remaining between-community level variance reduced by 46.7% and 11.4%, respectively. However, the between-community variance remained statistically significant. This demonstrates that the variables accounted for in the model do not unequivocally explain all the between-community variation in uptake of skilled birth care. In addition, the results presented in Table 2 shows that uptake of skilled birth care varies significantly between communities where CHPS has become functional within 8 km, even after adjusting for the important predictors in the models.

## Discussion

This study is the first of its kind to assess distance to facility as a key geographical barrier to the uptake of skilled birth care focusing on a government initiative that places Community Health Officers in rural communities. The results show that a substantial proportion of births continue to occur in communities with no access to both health facilities and CHPS, confirming poor access to healthcare in rural communities [8]. Although there has been a sharp rise in the number of CHPS compounds and facilities recently [13], the results show that access to CHPS facilities at birth until 2008 was very low.

The bivariate analysis shows that skilled birth care use in general has increased in communities where CHPS have become functional. Accounting for the important predictors, the multivariate analysis revealed that in communities where CHPS has become fucntional within 8 km but access to health facilities remain limited, the increase in uptake of skilled birth care are not large enough to be significant. Nonetheless, the results shows that there has been a significant increase in uptake of skilled birth care in communties where health facilities have been supplemented with CHPS within 8 km. Unsurprisingly, uptake of skilled birth care is significantly higher in communities with access to conventional health facilities compared to communities where there is no facility whatsoever within 8 km. More interesting is the finding that uptake of skilled birth care is significantly higher in communities where CHPS have become functional in combination with the existence of conventional health facilities when compared to communities with access to CHPS alone, or no access to both health facilities and CHPS.

The above findings demonstrate clearly that the CHPS programme alone has not significantly improved uptake of skilled birth care in rural communities of Ghana, but that the programme has been an effective supplement to the existing conventional facilities. Clearly, CHPS compounds were not really set up to deal with births themselves, so the finding that CHPS alone do not have an impact on SBA is not surprising. But the finding that CHPS facilitates use of skilled birth care at local facilities is encouraging. Although a significant association cannot prove the mechanism by which the effect is seen, a reasonable assumption is that CHPS staff have been active in referring women to facilities, or at least encouraging them to access more skilled care at conventional facilities. In this respect, the CHPS programme has been a significant catalyst for increased access to care at birth for rural women in Ghana.

It has to be noted that although provision of skilled care at birth is not specifically stated in the operational policy, CHPS has been repositioned as a major government policy instrument to address child and maternal health problems in the country, including the high rates of neonatal and maternal mortality [16, 17]. Research evidence shows that skilled birth care is essential in addressing the high rates of child and maternal mortality and morbidity rates in low and middle income countries. For example, a systematic review by showed that skilled birth care has the potential to reduce neonatal mortality by 25% and the provision of basic emergency obstetric care and comprehensive EmONC care can reduce these deaths by 40% and 85%, respectively [15]. Given the slow progress in uptake of skilled birth care in rural Ghana, it is vital that skilled care at birth is incorporated in the CHPS operational policy and action taken to ensure that women in CHPS zones where conventional health facilities are lacking have access to skilled maternity care including birthing care.

This study has shown that the CHPS initiative in its present structural orientation does not, on its own, promote skilled birth care use in rural communities. The Ghana MAF and Country Action Plan identified human resource challenges in CHPS zones, which could explain the low uptake of skilled maternity care in marginalised rural communities where CHPS are functional [7]. Evidence from the Upper East Region of Ghana shows that quality and uptake of skilled care at birth improved significantly in CHPS zones where the Ghana Health Services piloted an enhancement scheme by training CHPS Community Health Officers in midwifery skills [41]. Evidently, it is imperative that CHPS Community Health Officers should be trained and resourced to provide skilled delivery care within the communities they serve, particularly where access to health facilities are constrained.

Presently, the structual set-up of CHPS is such that all maternal health cases are referred to Health Centres. Although the CHPS initiative could help strengthen referral systems to address obstetric emergencies, this study demonstrates clearly that there are communities where CHPS has become functional but access to health facilities remain limited within 8 km, potentially weakening the programme’s impact. The substantial expansion of the CHPS initiative since 2009 is a step in the right direction, and our study shows that as a supplementary initiative to the main health system, the programme has acted as an effective additional force in widening access for rural women. However, effort should be made to further improve access to health facilities to strengthen the impact of the CHPS initiative.

Considering those factors that failed to show a significant effect is arguably one of the most interesting aspects of the analysis. For example, the fact that type of facility and EmONC status of the nearest facility are not significantly associated with uptake of skilled birth care is a really important finding. Clearly, the general public is not aware of the functioning status of health facilities, but studies from many other countries have shown that uptake can be related to perceived notions of quality. In the case of Ghana, a partially functioning EMONC status usually means that only one or two of the basic signal functions are missing, and indeed the facility may seem to provide enough services for women and their families to have enough confidence to access them.

With regards to communities where CHPS has become functional, irrespective of whether there is a health facility in close proximity or not, the results show that the number of antenatal visits has a significant positive effect on skilled birth care use, suggesting that contact with health services during pregnancy, whether because of complications or for routine checks, leads to a higher likelihood of giving birth with a skilled health worker. This is a common finding and confirms previous studies in Ghana and elsewhere [42]. Nontheless, in communities where CHPS facilities exist within 8 km but access to conventional health facilities remains limited, household wealth status is a significant predictor of access to skilled care, but this is not the case in communities where CHPS has supplement health facilities. This suggests that CHPS when implemented near to existing services, promotes equitable care that is accessible to both rich and poor alike. The results further shows that in communities where CHPS are functional, maternal age, education, religion, parity and region are not significant predictors of skilled birth care use, which suggests that in communities where CHPS are functional the effect of proximity to services outweighs most socioeconomic factors except for wealth status.

Our findings suggest that there is no substitute for high quality professional care and that the role of CHPS is as much about encouraging people into the system as providing care directly. The implications are that the government should examine carefully the geographical location not only of the CHPS service, but also of its primary health care system, and seek ways to optimise the relation and connectivity between the two.

A limitation of the study worth noting is that it is based on observational and not experimental data. Therefore, a direct causal relationship cannot be inferred between proximity to CHPS and uptake of skilled birth care. Also, there is no information on when conventional health facilities became functional within the communities in which they are located. Therefore, we are not able to establish explicitly the before and after impact of the functionality of conventional health facilities on the uptake of skilled birth care explicitly. However, given that the retrospective birth data refer to the functionality of the CHPS compounds and adjusting for their proximity to the communities, if the availability of CHPS compounds had any measurable impact then this should reflect in the uptake of skilled birth care. In addition, proximity to convensional health facilities and CHPS referal points were measured as the road network distance from the centroid of PSUs and not from residence of mothers, as the GDHS does not collect information on location of women. However, these effects are believed to be trivial since PSUs are census EAs with optimum population of 750 persons and represents local communities and is the smallest geographic units in the country [43]. Demarcation of EAs is also based on population density, terrain and for ease of enumeration they are geographically compact as possible [43].
